# Extracellular Signals of a Human Epithelial Colorectal Adenocarcinoma (Caco-2) Cell Line Facilitate the Penetration of *Pseudomonas aeruginosa* PAO1 Strain through the Mucin Layer

**DOI:** 10.3389/fcimb.2017.00415

**Published:** 2017-09-21

**Authors:** Naoki Hayashi, Atsushi Yokotani, Masami Yamamoto, Mariko Kososhi, Mayu Morita, Chiaki Fukunishi, Nagisa Nishizawa, Naomasa Gotoh

**Affiliations:** Department of Microbiology and Infection Control Science, Kyoto Pharmaceutical University Kyoto, Japan

**Keywords:** *Pseudomonas aeruginosa*, human colorectal adenocarcinoma cell line, flagellar motility, chemotaxis, translocation, sepsis, mucin, growth regulated oncogene-α

## Abstract

*Pseudomonas aeruginosa* can penetrate the layer of mucus formed by host intestinal epithelial cells, often resulting in sepsis in immunocompromised patients. We have previously demonstrated that *P. aeruginosa* can penetrate the mucin layer by flagellar motility and the degradation of the mucin layer. However, it remains unclear how *P. aeruginosa* initially recognizes epithelial cells. Using the human epithelial colorectal adenocarcinoma (Caco-2) cell line, we investigated extracellular signaling that could facilitate the penetration of *P. aeruginosa* through the mucin layer. The supernatant from Caco-2 cell cultures increased penetration of *P. aeruginosa* through an artificial mucin layer. The Caco-2 cell supernatant increased bacterial flagella-dependent swarming motility, but it did not influence *P. aeruginosa* growth or protease activity. Filtering of the Caco-2 cell supernatant indicated that proteins weighing <10 kDa increased mucin penetration, swarming motility, and, based on a tethered cell assay, induced acceleration of the flagellar filament rotational rate. Furthermore, a capillary assay showed that <10 kDa proteins in the Caco-2 cell supernatant attracted *P. aeruginosa* cells. Finally, we identified that growth-regulated oncogene-α (GRO-α) secreted by Caco-2 cells was a factor facilitating flagellar filament rotation and swarming motility, although it did not attract the bacteria. We conclude that penetration of the mucin layer by *P. aeruginosa* is facilitated by small proteins (<10 kDa) secreted by Caco-2 cells, both by inducing acceleration of flagellar motility and increasing chemotaxis.

## Introduction

The translocation of pathogens from the gut into the blood requires them to cross at least two barriers: the mucus layer and the tight junctions formed by epithelial cells (Dharmani et al., 2009; Tlaskalova-Hogenova et al., 2011). The mucus layer, which mainly comprises mucin protein, forms the first barrier between gut contents and epithelial cells (Dharmani et al., 2009). The epithelial cell layer, reinforced by tight junctions between the cells, forms the second barrier between the host and the environment (Tlaskalova-Hogenova et al., 2011). Moreover, host defenses include complex mechanisms of the innate immune system, such as phagocytosis by macrophages, dendritic cells, and neutrophils. These cells are attracted to the infection site by chemokines and play an important role in removing pathogens (Janeway and Medzhitov, 2002; Gellatly and Hancock, 2013; Sallenave, 2014). The Gram-negative bacterium *Pseudomonas aeruginosa* is a major opportunistic pathogen that causes severe infections, such as pneumonia and bacteremia, in immunocompromised patients. Both clinical (Ohara and Itoh, 2003; Shimizu et al., 2006; Vincent et al., 2009) and basic (Koh et al., 2010; Markou and Apidianakis, 2014) research suggests that the gastrointestinal tract is one of the reservoirs for *P. aeruginosa* in immunocompromised patients. Better understanding of opportunistic *P. aeruginosa* infections requires clarification of the mechanisms by which it penetrates the protective barriers and escapes the immune system defenses.

We consider the penetration of *P. aeruginosa* through the epithelial tissue to be at least a five-step process involving the following aspects: (i) recognition of epithelial cells, (ii) access to epithelial cells, (iii) adhesion to epithelial cells, (iv) formation of a permeation route, and (v) migration to a basolateral site (Okuda et al., 2010; Hayashi et al., 2013, 2015; Shikata et al., 2016). We have previously demonstrated that *P. aeruginosa* can penetrate the mucin layer by flagellar motility and mucin degradation (Hayashi et al., 2013). We subsequently showed that the type III effector molecule ExoS facilitates *P. aeruginosa* penetration through the epithelial cell layer by impairing the function of tight junctional proteins in their defense against bacterial penetration (Okuda et al., 2010). We recently showed that injection of ExoS into epithelial cells by *P. aeruginosa* is required for type IV pilus retraction (Hayashi et al., 2015; Shikata et al., 2016). However, the initial step of the entire process, that is how *P. aeruginosa* recognizes the epithelial cells in the first place, remains unclear.

*P. aeruginosa* flagellar motility, either swarming or swimming, derives from the rotation of the flagellar filament, which mainly comprises the major flagellum subunit protein FliC (Rashid and Kornberg, 2000; Macnab, 2003). The flagellar motor complex, MotAB and MotCD, provides energy for the rotational torque of the filament, which acts as a propeller (Doyle et al., 2004; Toutain et al., 2005). Deletion of the type IV pilus filament gene (*pilA*) increased spreading on swarming agar (Murray and Kazmierczak, 2008). Chemotaxis is required for flagellar filament rotation, and this motility allows *P. aeruginosa* to seek high levels of nutrients or to escape toxic compounds (Kato et al., 2008; Sampedro et al., 2015). Moreover, most *P. aeruginosa* isolates are motile, but even non-motile isolates can cause lethal endogenous bacteremia in leukopenic mice (Hatano et al., 1996). In the present study, we used a human epithelial colorectal adenocarcinoma (Caco-2) cell line to examine how *P. aeruginosa* recognizes mucosal epithelial cells so that it can then penetrate through the mucin layer.

## Materials and methods

### Bacterial strains, plasmids, and growth conditions

Our laboratory stock strains of *P. aeruginosa* are wild-type PAO1 (Stover et al., 2000), Δ*fliC* (Hayashi et al., 2013), Δ*motABCD* (Hayashi et al., 2013), and Δ*pilA* (Okuda et al., 2013). A green fluorescent protein (GFP) plasmid was constructed by subcloning a 700-bp *EcoR*I digested fragment containing the *gfp* gene derived from pGreen (Miller and Lindow, 1997) into the *EcoR*I site of pME6012 (Heeb et al., 2000). The resulting plasmid (pGFP) was then transfected into the wild-type PAO1 strain. *P. aeruginosa*, which were grown in Luria-Bertani (LB) broth (Nippon Becton Dickinson Company, Tokyo, Japan) or on LB agar plates (Nippon Becton Dickinson Company) at 37°C and supplemented with 50 μg/ml tetracycline (Sigma-Aldrich Co., St. Louis, MO, USA) when necessary.

### Preparation of Caco-2 cell supernatants

The Caco-2 cells were routinely grown at 37°C in a 95% air–5% CO_2_ atmosphere in Dulbecco's modified Eagle medium (DMEM) with high glucose (Sigma-Aldrich Co) and 10% heat-inactivated (56°C, 30 min) fetal bovine serum (FBS; Gibco, Grand Island, NY, USA). The cells were harvested using trypsin-EDTA (Gibco) and suspended with DMEM-10% FBS to stop the reaction with trypsin-EDTA. The cells were collected from the cell suspension by a centrifugation, and were washed with Dulbecco's phosphate buffered saline (DPBS; Gibco). Then, the cells were resuspended with DMEM without any serum, and the mixture was centrifuged twice. Finally, the cellular suspension in DMEM was adjusted to 1.0 × 10^5^ cells per 75 cm^2^ of the tissue culture flask (Thermo Scientific, Waltham, MA, USA). The culture medium was replaced every 5 days, and the supernatant collected after 5, 10, and 15 days of culture was used in subsequent experiments. The collected supernatant was filtered with Centriprep YM-3 or YM-10 (3 or 10 kDa; Millipore, Bedford, MA, USA). For some experiments, 500 μl of the various Caco-2 cell supernatant samples were treated with 25 μl of Immobilized TPCK Trypsin (Thermo Fisher) for 1 h at 37°C; the Immobilized TPCK Trypsin was then removed by centrifugation (10,000 × g for 10 min).

### Mucin penetration assay

We undertook an artificial mucin penetration assay as described previously (Hayashi et al., 2013). Mucin chambers were prepared by adding 50 μl of 3% bovine submaxillary mucin (Sigma-Aldrich Co) in DMEM to Transwell filter units containing 0.143 cm^2^ porous filter membranes (3.0-μm pores; Corning, New York, NY, USA). Next, 25 μl of *P. aeruginosa* culture (5.0 × 10^4^ colony-forming units; CFU) was pipetted onto the top of the mucin layers and 235 μl aliquots of the Caco-2 cell supernatant samples or DMEM as a control were placed in the bottom chambers of Transwell. After incubation for 3, 4, or 5 h, the bacteria were collected from the bottom chamber. Appropriate dilutions were spread on LB agar plates, incubated at 37°C overnight, and CFUs were counted to quantify the bacteria.

### Swarming assay

The bacterial motility swarming assay was modified from a previously reported method (Rashid and Kornberg, 2000) with the various Caco-2 supernatant samples (final: diluted to 50% with water) containing 0.5% (w/vol) bacto agar (Nippon Becton Dickinson Company) being used to assess swarming motility. A culture of the *P. aeruginosa* PAO1 strain was spotted on the center of the agar plate. The plate was incubated at 37°C in 5% CO_2_ for 15 h. After incubation, the radial distance (mm) from the center of the agar was measured.

### Measurement of bacterial growth

The *P. aeruginosa* PAO1 strain was incubated in the indicated samples at 37°C in 5% CO_2_. The optical density (600 nm) was measured at the indicated times. The optical density under each condition was analyzed.

### Measurement of protease activity

The assay was modified from a previously reported method (Kessler et al., 1982). Briefly, the *P. aeruginosa* PAO1 strain was incubated in the indicated samples at 37°C in 5% CO_2_ for 5 h. After incubation, 50 μl of the culture supernatant was added to 1.0 ml of 0.3% azocasein (Sigma-Aldrich Co) in a solution containing 5 mM Tris-HCI and 0.5 mM CaCl_2_ (pH 7.5). The reaction mixture was incubated at 37°C for 30 min. Undigested substrate was precipitated with 3.3% trichloroacetic acid and removed by centrifugation (10,000 × g for 10 min). The absorbance (440 nm) of the supernatant was measured. The absorbance under each condition was analyzed.

### Tethered cell assay

The assay was modified from a previously reported method (Qian et al., 2013). A glass slide was coated with Protein A (Biovision, Mountain View, CA, USA) for 30 min and washed with Dulbecco's phosphate buffered saline (DPBS; Gibco) to remove non-adherent Protein A. Thereafter, the slide was coated with anti-FliC flagellar antibody (Hayashi et al., 2013) for 30 min and washed with DPBS. The *P. aeruginosa* wild-type PAO1 strain was loaded onto the coated slide and non-tethered cells were rinsed off with DPBS. A cover slip was placed on the slide and the bacterial cells were visualized and recorded as a movie using a microscope with a 100 × objective for recording videos of tethered bacteria (EVOS; Thermo Fisher). Rotational speed was calculated from the number of rotations in 60 s.

### Chemotaxis assay

We undertook a capillary assay as described previously (Liu and Parales, 2008). A culture of the *P. aeruginosa* PAO1 strain with pGreen, expressing GFP, was centrifuged (1,800 × g for 5 min) and washed once with chemotaxis buffer (10 mM potassium phosphate buffer [pH 7.0], 0.1 mM disodium EDTA). One-microliter capillary tubes were filled with the indicated samples. The accumulation of cells in the capillary tip was observed using a fluorescence microscope with a 4 × objective (IX-71; Olympus, Tokyo, Japan). GFP intensities of the photograph were measured using ImageJ software (NIH, Bethesda, MD, USA).

### Measurement of cytokine concentrations

The concentration of cytokines in supernatant from Caco-2 cells cultured were determined by using the Multi-Analyte ELISArray Kits (Qiagen, Valencia, CA, USA) and Human GRO alpha (CXCL1) Platinum ELISA (Affymetrix, Santa Clara, CA, USA) by following the manufacturer's instructions.

### Chemokines and antibody

Recombinant human chemokines, including interleukin-8 (IL-8); regulated on activation, normal T cell expressed and secreted (RANTES); macrophage inflammatory protein-1α (MIP1α); macrophage-derived chemokine (MDC); and growth regulated oncogene-α (GRO-α) were purchased from PeproTech Inc. (Rocky Hill, NJ, USA). An anti-GRO-α antibody was purchased from Abcam Plc (Cambridge, MA, USA), and normal goat serum was purchased from Wako Pure Chemical Industries Ltd. (Osaka, Japan).

### Statistical analysis

Statistical analysis, including one-way analysis of variance (ANOVA), Tukey's test, and Student's *t*-test, was performed with EZR (Kanda, 2013), a graphical user interface for R. More precisely, it is a modified version of the R commander designed to add statistical functions frequently used in biostatistics.

## Results

### Caco-2 cell culture supernatant facilitates the penetration of *P. aeruginosa* through the mucin layer

**Figure 1:**
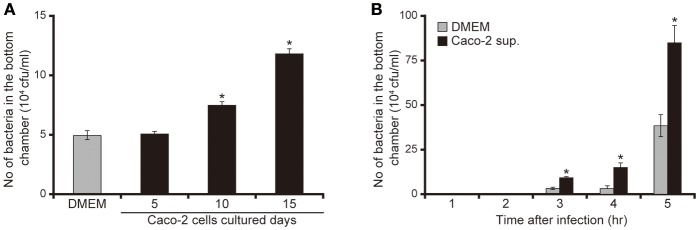
Caco-2 cell supernatant facilitates penetration of the *P. aeruginosa* PAO1 strain through the mucin layer. **(A)** The bottom chambers of Transwell cells were filled with Caco-2 cell supernatant (collected after 5, 10, and 15 days of culture) or with Dulbecco's modified Eagle medium (DMEM; control). The *P. aeruginosa* PAO1 strain was added to the top chamber. After 3 h, the numbers of bacteria in the bottom chambers were counted. Error bars indicate standard error (*n* = 3). ^*^P < 0.05 compared with DMEM. **(B)** Caco-2 cell supernatant after 15 days of culture (Caco-2 sup.) or DMEM (control) filled the bottom chamber of Transwell cells. After the *P. aeruginosa* PAO1 strain was added, the numbers of bacteria in the bottom chambers were counted at 3, 4, and 5 h. Error bars indicate standard error (*n* = 3). ^*^P < 0.05 compared with DMEM at the same incubation time.

To clarify the mechanism by which *P. aeruginosa* recognizes extracellular signals of the intestinal epithelial cells using the human intestinal epithelial Caco-2 cell line (Okuda et al., 2010; Hayashi et al., 2013, 2015; Shikata et al., 2016), we examined the effects of Caco-2 cell culture supernatants on mucin penetration by the *P. aeruginosa* PAO1 strain. After 3 h of incubation, the bacterial numbers in the bottom chamber of wells with 10- and 15-day Caco-2 cell supernatant were 1.6 and 2.5 times higher than those with DMEM, respectively (Figure 1A; *P* < 0.05). The effect of the 5-day supernatant on penetration did not differ significantly from that of DMEM (Figure 1A; *P* > 0.10). After 3, 4, and 5 h of incubation, the numbers of bacteria in the bottom chambers filled with 15-day supernatant were 2.5, 2.2, and 2.3 times greater than those cultured with DMEM, respectively (Figure 1B; *P* < 0.05).

### Characteristic of Caco-2 cell signaling facilitating the penetration of *P. aeruginosa* through the mucin layer

**Figure 2:**
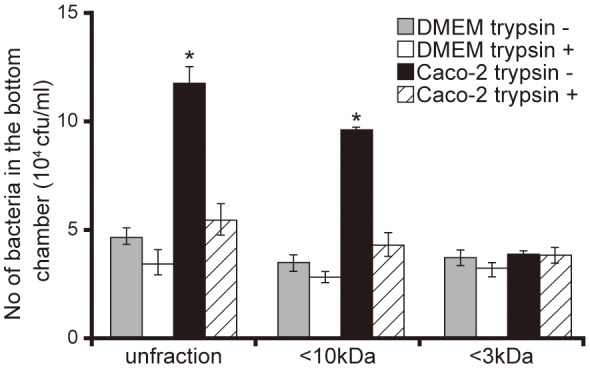
Characterization of Caco-2 cell supernatant facilitating penetration of *P. aeruginosa* PAO1 strain through the mucin layer. A 15-day Caco-2 cell supernatant and Dulbecco's modified Eagle medium (DMEM; control) were filtered through 10- or 3-kDa MW cut-off membranes and treated with or without a trypsin (trypsin + or −). The filtered and unfiltered samples with or without trypsin filled the bottom chambers of Transwell. The *P. aeruginosa* PAO1 strain was added to the top chambers, and after 3 h, the numbers of bacteria in the bottom chambers were counted. Error bars indicate standard error (*n* = 3). ^*^P < 0.05 compared with DMEM of the same fraction size.

To characterize Caco-2 cell signals that facilitated the penetration of *P. aeruginosa* through the mucin layer, we performed an artificial mucin layer penetration assay using filtered and/or trypsin-treated 15-day Caco-2 cell supernatants. The bacterial numbers in bottom chambers of Transwell filled with unfiltered and <10 kDa Caco-2 cell supernatant fractions were 2.5 and 2.7 times higher than those with DMEM of the same fraction size (Figure 2; *P* < 0.05). The number of bacteria penetrating the mucin layer did not differ significantly between those treated with <3 kDa Caco-2 cell supernatant and the DMEM control (Figure 2; *P* > 0.10). Moreover, trypsin treatment of unfiltered and the <10 kDa Caco-2 cell supernatant fraction was associated with less *P. aeruginosa* penetration through the mucin layer (Figure 2).

### Caco-2 cell supernatant facilitates swarming motility of *P. aeruginosa*

**Figure 3:**
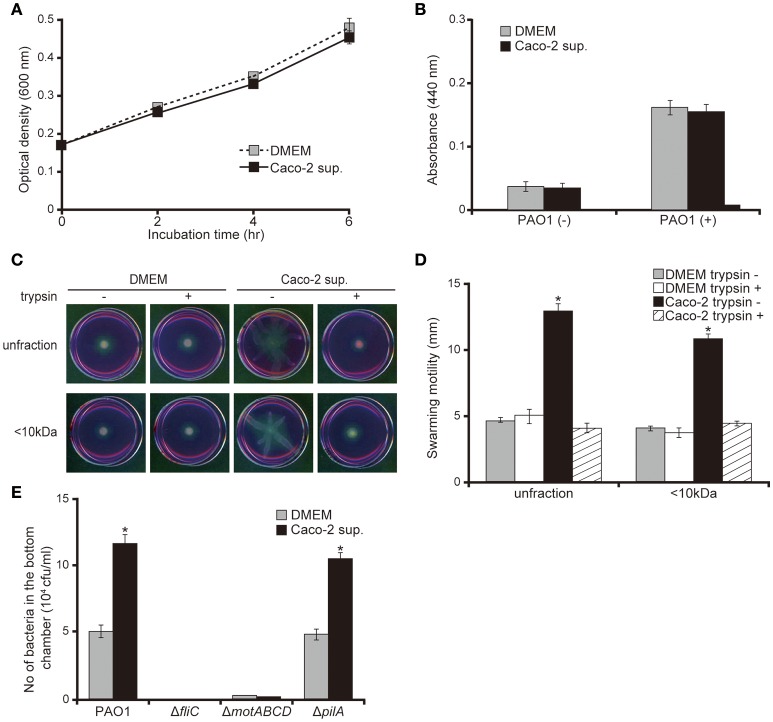
Caco-2 cell supernatant facilitates a swarming motility of the *P. aeruginosa* PAO1 strain. **(A)** The *P. aeruginosa* PAO1 strain was incubated in Caco-2 cell supernatant (Caco-2 sup.) or Dulbecco's modified Eagle medium (DMEM; control) at 37°C in 5% CO_2_. After incubation, the optical density (600 nm) of the culture was measured at the indicated times. Error bars indicate standard error (*n* = 3). **(B)** After incubation of the *P. aeruginosa* PAO1 strain in Caco-2 cell supernatant (Caco-2 sup.) or DMEM (control) at 37°C in 5% CO_2_ for 5 h, the azocasein degradation activity of the collected culture supernatant was measured. Error bars indicate standard error (*n* = 3). **(C)** A 15-day Caco-2 cell supernatant (Caco-2 sup.) and DMEM (control) were filtered through 10-kDa MW cut-off membranes. The <10 kDa fraction and unfiltered samples were treated with or without trypsin (trypsin + or −). The *P. aeruginosa* PAO1 strain was spotted on swarming agar containing the treated samples. After incubation for 14 h, the swarming agar plates were observed and photographed. **(D)** The radial distance (mm) from the center of the agar was measured. Error bars indicate standard error (*n* = 3). ^*^P < 0.05 compared with DMEM at the same size fraction. **(E)** A 15-day Caco-2 cell supernatant (Caco-2 sup.) or DMEM (control) was filled in the bottom chamber of Transwell. The *P. aeruginosa* PAO1 strain, flagellar mutants (Δ*fliC* and Δ*motABCD*), or a type IV pilus mutant (Δ*pilA*) were added to the top chambers, and after 3 h, the numbers of bacteria in the bottom chambers were counted. Error bars indicate standard error (*n* = 3). ^*^P < 0.05 compared with incubation in DMEM of the same strains.

No significant difference was observed in bacterial growth and protease activity between bacteria incubated with Caco-2 cell supernatants or the DMEM control (Figures 3A,B; *P* > 0.10). Both unfiltered Caco-2 cell supernatant and the filtered <10 kDa fraction significantly increased the swarming motility of the *P. aeruginosa* PAO1 strain by 2.7 times (Figures 3C,D; *P* < 0.05). Trypsin treatment of the supernatant diminished the increase in motility on the swarming agar by the Caco-2 cell supernatants (Figures 3C,D). *P. aeruginosa* has two major structures that contribute to motility: the flagella and type IV pili. Deletion of a type IV pilus filament gene (*pilA*) increased the motility on the swarming agar (Murray and Kazmierczak, 2008). No significant difference was observed in terms of the numbers of either the wild-type PAO1 strain or a mutant with type IV pilus filament deletion (the Δ*pilA* strain) penetrating the mucin layer when treated with either DMEM or Caco-2 cell supernatant (Figure 3E; *P* > 0.10). Penetration of mutants with flagellar filament deletion (the Δ*fliC* strain) and motor deletion (the Δ*motABCD* strain) through the mucin layer could not be detected at 3 h after inoculation (Figure 3E).

### Caco-2 cell supernatant facilitates rotation of the *P. aeruginosa* flagellar filament

**Figure 4:**
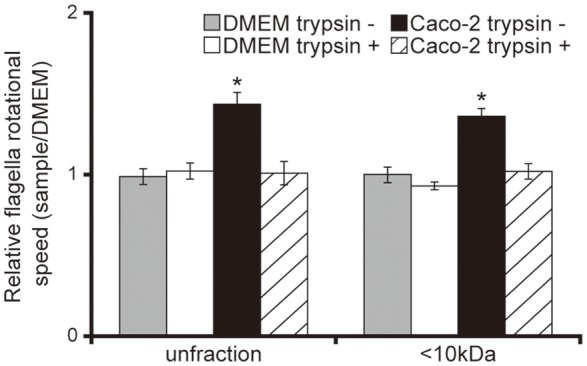
Caco-2 cell supernatant facilitates a flagellar filament rotation of the *P. aeruginosa* PAO1 strain. A 15-day Caco-2 cell supernatant and Dulbecco's modified Eagle medium (DMEM; control) were filtered through 10-kDa MW cut-off membranes. The <10 kDa fraction or unfiltered samples were treated with or without trypsin (trypsin + or −). *P. aeruginosa* PAO1 strain cells treated with the various samples were loaded onto a glass slide precoated with flagellar filament FliC antibodies. Bacterial cells were visualized and recorded as a movie using EVOS microscope, showing videos of tethered bacteria. The rotational speed was calculated from the number of rotations in 60 s. Error bars indicate standard error (*n* = 10). ^*^P < 0.05 compared with DMEM of the same fraction size.

Having demonstrated that <10 kDa proteins secreted by Caco-2 cells induced the acceleration of flagellar motility in the *P. aeruginosa* PAO1 strain, thus facilitating penetration of the mucin layer (Figures 1–3), we sought to clarify the mechanism with a tethered cell analysis, measuring the rotational speed of flagellar filaments. The flagellar filament rotational speed of the *P. aeruginosa* PAO1 strain treated with unfiltered and the <10 kDa fraction of the Caco-2 cell supernatant was 1.4 times greater than with DMEM at the same fraction size (Figure 4; *P* < 0.05). Trypsin treatment of the Caco-2 cell supernatant diminished the increased rotational speed (Figure 4). No significant differences were observed in the number of bacteria adhering to glass slides under all conditions (data not shown).

### Caco-2 cell supernatant attracts the *P. aeruginosa* PAO1 strain

**Figure 5:**
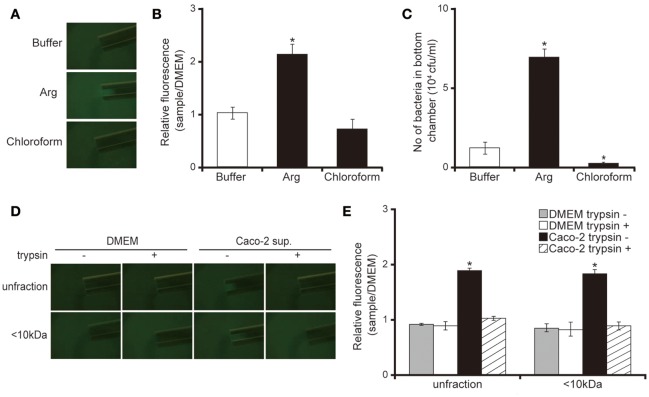
Caco-2 cell supernatant attracts the *P. aeruginosa* PAO1 strain. **(A)** A glass slide surface was filled with a *P. aeruginosa* PAO1 strain expressing a green fluorescent protein (GFP). Capillary tubes were filled with buffer (control), Arg (attractant), or chloroform (repellent). The accumulation of the *P. aeruginosa* PAO1 strain expressing GFP at the capillary tip was visualized and photographed by microscopy at a magnification of 40×. **(B)** The graph shows the relative intensity of GFP in the capillary after incubation for 10 min. Error bars indicate standard error (*n* = 3). ^*^P < 0.05 compared with buffer. **(C)** A buffer (control), Arg (attractant), or chloroform (repellent) filled the bottom chamber of Transwell. The *P. aeruginosa* PAO1 strain was added and, after 3 h, the numbers of bacteria in the bottom chambers were counted. Error bars indicate standard error (*n* = 3). ^*^P < 0.05 compared with buffer. **(D)** A 15-day Caco-2 cell supernatant and Dulbecco's modified Eagle medium (DMEM; control) were filtered through 10-kDa MW cut-off membranes. The <10 kDa fraction or unfiltered samples were treated with or without trypsin (trypsin + or −). A cover glass surface was filled with the *P. aeruginosa* PAO1 strain expressing GFP. Capillary tubes were filled with the treated samples. The accumulation of the GFP-expressing *P. aeruginosa* PAO1 strain at the capillary tip was visualized and photographed by microscopy at a magnification of 40×. **(E)** The graph shows the relative intensity of GFP in the capillary tube after incubation for 10 min. Error bars indicate standard error (*n* = 3). ^*^P < 0.05 compared with DMEM of the same fraction size.

Our capillary assay showed that 10 mM of L-arginine attracted and chloroform repelled *P. aeruginosa* cells (Figures 5A,B; *P* < 0.05). These data were consistent with previous findings (Craven and Montie, 1985; Shitashiro et al., 2003; Kato et al., 2008). To examine whether the chemoattractant facilitates penetration of *P. aeruginosa* through the mucin layer, we added the chemoattractant or repellent to the bottom chamber of Transwell. After 3 h of incubation, 10 mM L-arginine significantly increased the number of *P. aeruginosa* in the bottom chamber (Figure 5C; *P* < 0.05). In contrast, chloroform diminished the number of *P. aeruginosa* in the bottom chamber (Figure 5C; *P* < 0.05). A good correlation was observed between chemotaxis and the penetration of the *P. aeruginosa* PAO1 strain through the mucin layer. Thereafter, to clarify whether the Caco-2 cell supernatant has the ability to attract *P. aeruginosa*, we performed a capillary assay. Both the unfiltered and the <10 kDa Caco-2 cell supernatant fraction increased GFP intensity in the capillary tubes (Figures 5D,E; *P* < 0.05). Trypsin treatment of the Caco-2 cell supernatant diminished this increased GFP intensity in the capillary tube (Figures 5D,E; *P* < 0.05).

### Cytokines secreted by Caco-2 cells facilitate the penetration of the *P. aeruginosa* PAO1 strain through the mucin layer

**Figure 6:**
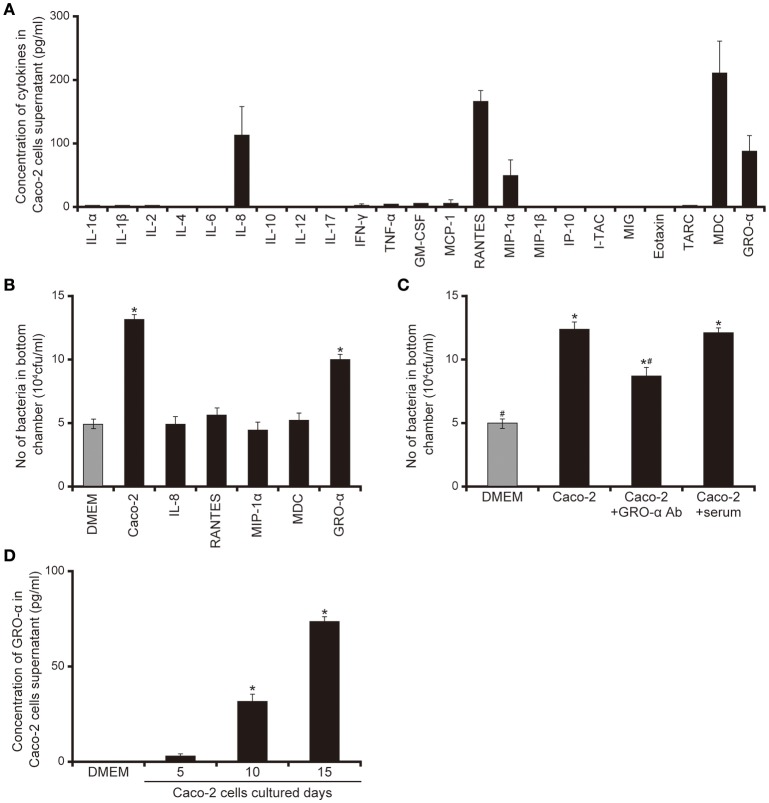
Chemokines secreted by Caco-2 cells facilitate the penetration of the *P. aeruginosa* PAO1 strain through the mucin layer. **(A)** Cytokine levels in the 15-day Caco-2 cell supernatant were determined by enzyme-linked immunosorbent assay (ELISA). The graph shows the cytokine concentrations in the supernatant. Error bars indicate standard error (*n* = 3). **(B)** The cytokines identified by the assay were placed in the bottom chamber of Transwell, as were unfiltered 15-day Caco-2 cell supernatant and Dulbecco's modified Eagle medium (DMEM; control). The *P. aeruginosa* PAO1 strain was added to the top chambers and, after 3 h, the numbers of bacteria in the bottom chamber were counted. Error bars indicate standard error (*n* = 3). ^*^P < 0.05 compared with DMEM. **(C)** Caco-2 cells supernatant were pretreated for 30 min with anti-GRO-α antibodies (GRO -α Ab) (1 ml of Caco-2 cells supernatant/100 ng of antibody) or normal goat serum (serum) then inoculated onto the bottom chamber of Transwell. Error bars indicate standard error (*n* = 3). ^*^P < 0.05 compared with DMEM. ^#^*P* < 0.05 compared with Caco-2 cell supernatant. ^*^^#^*P* < 0.05 compared with DMEM and *P* < 0.05 compared with Caco-2 cell supernatant. **(D)** Growth regulated oncogene-α concentrations in the 5-, 10-, and 15-day Caco-2 cell supernatants were determined by ELISA. Error bars indicate standard error (*n* = 3). ^*^P < 0.05 compared with DMEM.

Our data suggested that smaller proteins secreted by Caco-2 cells induced the acceleration of flagellar motility required for penetration through the mucin layer (Figures 1–5). We therefore measured various cytokines secreted from Caco-2 cells using enzyme-linked immunosorbent assay (ELISA). As shown in Figure 6A, five cytokines were detected in the 15-day Caco-2 cell supernatant (Figure 6A), IL-8 (112.2 pg/mL), RANTES (162.9 pg/mL), MIP1α, (45.4 pg/mL), MDC (221.3 pg/mL), and GRO-α (83.4 pg/mL) (Figure 6A). No cytokines were detected in DMEM (data not shown). We subsequently investigated whether the chemokines in the Caco-2 cell supernatant could have facilitated the penetration of *P. aeruginosa* through the artificial mucin layer. We added each of the five chemokines at the concentrations detected in the Caco-2 cell supernatant to the bottom chambers of Transwell and assessed the penetration of *P. aeruginosa*. Of the five chemokines, only GRO-α significantly increased the number of bacteria, by 2.0-fold; the Caco-2 cell supernatant increased 2.3-fold (Figure 6B; *P* < 0.05). Furthermore, addition of anti- GRO-α antibodies into Caco-2 cells supernatant significantly decreased penetration through mucin layers (Figure 6C; *P* < 0.05). The effect of normal goat serum on penetration did not differ significantly from that of Caco-2 cells supernatant (Figure 6C; *P* > 0.10). GRO-α concentrations in the Caco-2 cell supernatant increased with the length of Caco-2 culture (Figure 6D; *P* < 0.05). A good correlation was observed between the increase of GRO-α concentration and that in mucin penetration by *P. aeruginosa* (Figures 1A, 6D).

### GRO-α facilitates flagellar motility of the *P. aeruginosa* PAO1 strain

**Figure 7:**
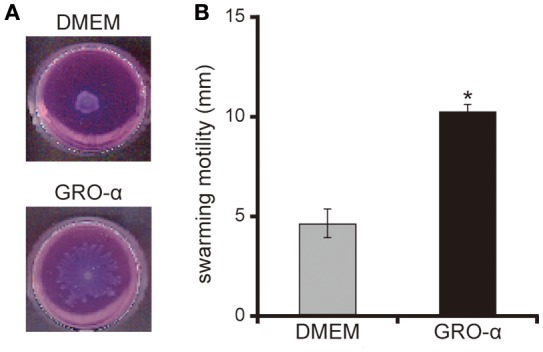
GRO-α facilitates a swarming motility of the *P. aeruginosa* PAO1 strain. **(A)** The *P. aeruginosa* PAO1 strain was spotted on swarming agar containing growth regulated oncogene -α or Dulbecco's modified Eagle medium (DMEM; control). After incubation for 14 h, the swarming agar plate was observed and photographed. **(B)** The radial distance (mm) from the center of the agar was measured. Error bars indicate standard error (*n* = 3). ^*^P < 0.05 compared with DMEM.

**Figure 8:**
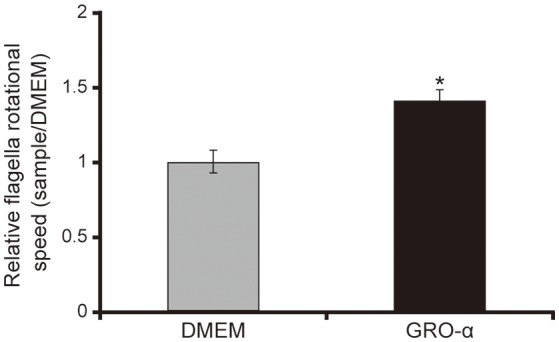
GRO-α facilitates a flagellar filament rotation of the *P. aeruginosa* PAO1 strain. *P. aeruginosa* PAO1 strain cells were loaded onto a glass slide precoated with flagellar filament FliC antibodies, and growth regulated oncogene-α or Dulbecco's modified Eagle medium (DMEM; control) was added. Bacterial cells were visualized and recorded as a movie using EVOS microscope, showing videos of the tethered bacteria. The rotational speed was calculated from the number of rotations in 60 s. Error bars indicate standard error (*n* = 10). ^*^P < 0.05 compared with DMEM.

**Figure 9:**
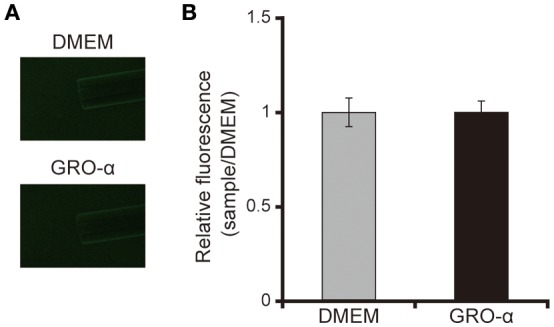
GRO-α does not attract the *P. aeruginosa* PAO1 strain. **(A)** A glass slide was filled with the GFP-expressing *P. aeruginosa* PAO1 strain. A capillary tube was filled with growth regulated oncogene-α or Dulbecco's modified Eagle medium (DMEM; control). The accumulation of *P. aeruginosa* at the capillary tip was visualized and photographed by microscopy at a magnification of 40×. **(B)** The graph shows the relative intensity of GFP in the capillary tube after incubation for 10 min. Error bars indicate standard error (*n* = 3).

The addition of GRO-α (83.4 pg/mL) to swarming agar led to a significant 2.3-fold increase in the swarming motility of the *P. aeruginosa* PAO1 strain (Figures 7A,B; *P* < 0.05). Next, we measured the rotational speed of the flagellar filament by a tethered cell analysis. GRO-α (83.4 pg/mL) led to a significant 1.4-fold increase in the flagellar filament rotation speed of the *P. aeruginosa* PAO1 strain (Figure 8; *P* < 0.05). No significant difference was observed between GRO-α (83.4 pg/mL) and DMEM control in the number of bacteria adhering to the glass slides (data not shown). Treatment with GRO-α did not show any difference in increased GFP intensity in capillary tubes (Figures 9A,B; *P* > 0.10).

## Discussion

In this study, we examined the effects of the supernatant of Caco-2 cells cultured *in vitro* on mucin penetration by the *P. aeruginosa* PAO1 strain to clarify the mechanism by which *P. aeruginosa* recognizes extracellular signals from intestinal epithelial cells to facilitate bacterial translocation. Our results indicated that the <10 kDa proteins secreted by Caco-2 cells facilitated the penetration of *P. aeruginosa* through the mucin layer. These small proteins enhanced swarming motility but did not influence the growth rate or protease activity of the *P. aeruginosa* PAO1 strain. We then found that the <10 kDa proteins secreted by Caco-2 cells accelerated flagellar filament rotation and attracted the *P. aeruginosa* PAO1 strain into capillary tubes. Among the tested chemokines, GRO-α was found to facilitate mucin layer penetration by *P. aeruginosa* by inducing the acceleration of flagellar motility, although not by increased chemotaxis. We conclude that GRO-α and other unknown <10 kDa proteins secreted by intestinal epithelial cells facilitate penetration of the mucin layer by *P. aeruginosa*.

We previously demonstrated that *P. aeruginosa* penetrates the mucin layer using flagellar motility and mucin degradation (Hayashi et al., 2013). The present study showed that Caco-2 cells enhanced flagella-dependent swarming motility but did not influence the growth rate or protease activity of the *P. aeruginosa* PAO1 strain. *P. aeruginosa* flagellar motility derives from rotation of the flagellar filament, which mainly comprises the major flagellum subunit protein FliC (flagellin) (Rashid and Kornberg, 2000; Macnab, 2003). Some researchers have suggested that three main factors can affect *P. aeruginosa* motility on swarming agar: (i) acceleration of flagellar filament rotation (Rashid and Kornberg, 2000; Macnab, 2003; Doyle et al., 2004; Toutain et al., 2005), (ii) addition of a chemoattractant to the swarming agar (Tremblay et al., 2007), and (iii) deficiency of type IV pili (Murray and Kazmierczak, 2008). As no significant difference was observed in the penetration of the *P. aeruginosa* pilus filament mutant (Δ*pilA* strain) through the mucin layers with or without the Caco-2 cell supernatant, it is suggested that the Caco-2 cell supernatant does not affect the function of the type IV pili in relation to flagellar motility. However, the Caco-2 cell supernatant did significantly accelerate flagellar filament rotation, as well as exhibit chemoattraction for the *P. aeruginosa* PAO1 strain. Thus, collectively, our novel findings suggest that Caco-2 cells facilitate *P. aeruginosa* penetration through the mucin layer by at least two mechanisms: acceleration of flagellar filament rotation and chemotaxis.

Our data showed that <10 kDa proteins secreted by Caco-2 cells facilitated the penetration of *P. aeruginosa* through the mucin layer, along with its swarming motility. Moreover, the supernatant filtrate containing these <10 kDa proteins had the same effect as the unfiltered supernatant on flagellar filament rotation and chemotaxis. The mucin penetration and swarming motility in addition of GRO-α alone in the same concentration in Caco-2 cell supernatant showed the 75 and 78% in addition of Caco-2 cell supernatant, respectively. Then, we found that GRO-α alone in the same concentration as that in the Caco-2 cell supernatant had an even greater effect than the supernatant alone on flagellar filament rotational speed. It did not, however, attract *P. aeruginosa* cells. Taken together, our data suggest that Caco-2 cells secrete several factors that affect *P. aeruginosa*. GRO-α, whether alone or along with other unidentified substances, facilitates flagellar filament rotation. However, there must also be one or more unknown small proteins serving as chemoattractants for *P. aeruginosa*. Further studies are necessary to identify other attractants secreted by Caco-2 cells.

Our data revealed that <10 kDa signals secreted by Caco-2 cells, including GRO-α and other unidentified ones, facilitated the flagellar motility. *P. aeruginosa* flagellar motility is derived from flagellar filament rotation by flagellar motor complexes, MotAB and MotCD (Rashid and Kornberg, 2000; Macnab, 2003; Doyle et al., 2004; Toutain et al., 2005), but previous reports about a receptor directly regulating the function of the flagellar motor complex in *P. aeruginosa* are absent. On the contrary, receptors regulating the chemotaxis of *P. aeruginosa* have been identified (Kato et al., 2008; Sampedro et al., 2015). Further studies are necessary to identify the receptors in *P. aeruginosa* for the signals secreted by Caco-2 cells.

Pathogenic bacteria possess virulence factors that facilitate escape from host defense mechanisms. The relation between cytokine production and the establishment of bacterial infection has been examined. Most reports, including investigations of *P. aeruginosa* infections, have focused on the systems by which host cells recognize pathogens or pathogens escape this immune surveillance (Janeway and Medzhitov, 2002; Gellatly and Hancock, 2013; Sallenave, 2014). In contrast, Wu et al. suggested that pathogens may actively respond to a host's immune signals. They showed that interferon-gamma binds to a *P. aeruginosa* outer membrane protein, OprF, resulting in the expression of PA-I lectin-dependent cytotoxicity directed toward epithelial cells (Wu et al., 2005). GRO-α has been shown to attract neutrophils to sites of infection (Bechara et al., 2007), but there have previously been no reports of GRO-α directly affecting *P. aeruginosa* virulence. A novel finding of our study is the role of GRO-α in promoting *P. aeruginosa* virulence by augmenting the bacterium's flagellar filament rotational speed, a factor that facilitates the penetration of *P. aeruginosa* through the mucin layer. Recently, Shieh et al. reported that human airway epithelial cells secrete GRO-α (Shieh et al., 2014). Therefore, GRO-α might induce the *P. aeruginosa* cystic fibrosis and pneumonia by mechanisms identical to those of our findings.

In summary, our study using *in vitro* intestinal epithelial cells showed that small proteins of <10 kDa secreted by these cells, including GRO-α and other as yet unidentified ones, may be important factors in the initiation of *P. aeruginosa* translocation. Further studies are required to clarify the relation between host cells and *P. aeruginosa*. Ultimately, understanding these mechanisms may lead to new therapeutic strategies for *P. aeruginosa* gut-derived sepsis.

## Author contributions

NH and NG designed this research. NH, AY, MY, MK, MM, CF, and NN performed experiments. NH and AY analyzed the data. NH and AY drafted the manuscript. All authors have read the manuscript and approved its submission.

### Conflict of interest statement

The authors declare that the research was conducted in the absence of any commercial or financial relationships that could be construed as a potential conflict of interest.
